# Ascorbic Acid and Glucosinolate Levels in New Czech Cabbage Cultivars: Effect of Production System and Fungal Infection

**DOI:** 10.3390/molecules23081855

**Published:** 2018-07-25

**Authors:** Cenek Novotny, Vera Schulzova, Ales Krmela, Jana Hajslova, Katerina Svobodova, Martin Koudela

**Affiliations:** 1 Laboratory of Environmental Biotechnology, Institute of Microbiology of the CAS, v.v.i., Vídeňská 1083, 142 20 Prague 4, Czech Republic; ksvobod@biomed.cas.cz; 2 Department of Food Analysis and Nutrition, Faculty of Food and Biochemical Technology Prague, University of Chemistry and Technology, Technická 5, 166 28 Prague 6, Czech Republic; vera.schulzova@vscht.cz (V.S.); ales.krmela@vscht.cz (A.K.); jana.hajslova@vscht.cz (J.H.); 3 Department of Horticulture, Faculty of Agrobiology, Food and Natural Resources, Czech University of Life Sciences Prague, Kamýcká 129, 165 21 Prague 6, Czech Republic; koudela@af.czu.cz

**Keywords:** head cabbage, ascorbic acid, glucosinolates, Albatros cultivar, target cultivar, integrated system, ecological system, fungal infection, *Alternaria brassicicola*

## Abstract

Nutritional value and disease-preventive effects of cabbage are well-known. Levels of the antioxidant compounds ascorbic acid (AA) and glucosinolates (GSL) in new Czech cabbage cultivars were determined in the context of different production systems. The contents of AA and GSLs in cabbage biomass were determined by HPLC. Individual GSLs were identified according to their exact masses with sinigrin used as the external standard. Artificial infection with *A. brassicicola* generally raised the AA levels. The major GSLs (≥10 mg kg^−1^) were glucobrassicin, sinigrin, and glucoiberin. Indole and aliphatic GSLs were present, but no aromatic ones were detected. Ecological growth conditions and the artificial fungal infection increased the total content of GSLs and, also, of the methoxylated indole GSLs. Sulforaphane, iberin, indole-3-carbinol, and ascorbigen resulting from the hydrolysis of GSLs were found in both cultivars. The amounts and profiles of GSLs present in the two Czech cultivars demonstrated their good nutritional value. The decomposition products sulforaphane, iberin, indole-3-carbinol, and ascorbigen detected improve its health-promoting qualities and represent a suitable component of the human diet.

## 1. Introduction

Head cabbage belongs to vegetables with a high nutrient-to-price ratio and contain phytochemicals associated with potential human health benefits. The beneficial effects have been attributed to the antioxidant-activity compounds such as ascorbic acid (AA) and glucosinolates (GSLs) [1,2]. Cabbage is an important source of AA and GSLs in the human diet.

Various cabbage cultivars have been shown to contain concentrations of AA ranging from 316 to 676 mg kg^−1^ [1,3,4]. They are influenced by the year, vegetation period, fertilization and geographical conditions [5,6]. The effect of fertilization on AA content is ambiguous; the use of green manure raised the amount of AA in cabbage whereas no effect was found when an NPK fertilizer [containing as macronutrients nitrogen (N), phosphorus (P) and potassium (K)] or compost were used [7,8,9].

GSL content varies between various plant species and cultivars, type of tissue, developmental stage, and the sulphur supply status of the plant [10,11,12,13,14]. GSLs accumulate in *Brassica* tissues as a result of various stresses or after infestation by pathogens. The spread of the infection and the development of subsequent infections are inhibited [15]. The accumulation of GSLs is defined by the magnitude and duration of the stress [16]. The main GSL side-chains occurring in cabbage are 2-propenyl-(sinigrin), 3-methylsulfinylpropyl-(iberin), and indolylmethyl-(glucobrassicin), and they are present at respective concentrations of 0.04–1.6, 0.05–2.6 and 0.09–2 mmol kg^−1^ fresh cabbage biomass. The range of concentrations in cabbage is similar to that in broccoli, Brussels sprouts and cauliflower [16,17,18]. The main indole GSL in cabbage is glucobrassicin, and the content of 4-methoxy-3-indolylmethyl GSL is two times lower [19].

Loss of the cellular integrity as a result of biological or abiotic stress leads to a hydrolysis of GSLs by the enzyme myrosinase. The products of GSL hydrolysis are toxic to bacteria and fungi [20,21]. A study with fungal pathogen *Leptosphaeria maculans* demonstrated a higher toxicity of aromatic ITC, compared to aliphatic ones, with the toxicity of the latter decreasing with the size of the side-chain [22]. Glucoerysolin was identified as the major active compound with a broad spectrum of antimicrobial activity [23].

The purpose was to compare the production of health-beneficial phytochemicals of new Czech cabbage Albatros and Target cultivars (cv.) with other cabbage varieties used for production in Europe, when produced in the integrated and ecological production systems. A further aim was to evaluate the cultivar resistance to the artificial infection with *A. brassicicola* and the effect of the infection on the contents of AA and GSLs

## 2. Results and Discussion

### 2.1. Effect of Production System on AA Contents

The type of production, integrated or ecological, together with other environmental conditions including pests, may significantly influence the consumer quality of the produced vegetables [24,25,26]. Table 1 summarizes the results showing the effect of the integrated, conventionally-grown and the ecological types of production on the content of AA and GSLs. In neither production system fungicides were applied to avoid suppression of *A. brassicicola* and spontaneous fungal infection.

The contents of AA in Albatros and Target cv. were 1713–4326 and 2684–3353 mg kg^−1^ dry biomass, respectively, depending on the production system (Table 1). Similarly, studies by other authors on cabbage and other vegetables such as carrot, onion, pea, and potato reported no significant differences dependent on the growing system used, but they concluded that the results can be season dependent [27,28]. AA contents measured in both cultivars (cf. Table 1) were similar to those of Polish’Stone head’cabbage [29], Spanish Hinova, Megaton, Alfredo, Candela, and Bronco cv. [6], green cabbage cv. [30], and Savoy cabbage cv. [31] but were slightly lower than those detected in *Brassica oleracea* L. var. *capitata* cv. Lennox produced under conventional or organic conditions [26].

The artificial infection with *A. brassicicola* was effective in both production systems as it resulted in a higher damage to Albatros and Target cv. compared to the noninfected controls (Table 1). Albatros was more resistant to the artificial and spontaneous infections than Target. The statistical analysis of the results that used ANOVA and Fisher’s LSD test is shown in Table 1. The fungal infection raised the quantity of AA in both varieties 1.3–2.5-fold irrespective of the production system (Table 1). This confirms the observation in cucumber where a pretreatment with *Trichoderma asperelloides* increased the total ascorbate content two times [32]. Similar activation of the antioxidant machinery was observed during artificial colonization of roots of *Arabidopsis*, cucumber, and tomato plants by *T. asperelloides* and *T. harzianum*. Such an activation resulted in an enhancement of tolerance to a range of abiotic stresses, e.g., salt stress or water deficit [32,33].

### 2.2. Contents of GSLs in Dependence on the Production System

Albatros cv. mostly exhibited higher levels of total GSLs, compared to Target cv. The differences did not exceed 20% (Table 1). Similarly, a 1.3-fold difference in the content of total GSLs was reported for Herfstraap and Oleifera cv. of *Brassica rapa* [34]. The range of total GSL content in Albatros and Target were comparable with numerous varieties of white cabbage (*B. oleracea* var. *capitata*), Romanian *Brassica* vegetables, and green cabbage [30,35,36]. Commercial samples of white cabbage (*B. oleracea* var. *capitata* f. *alba*) purchased in supermarkets in England, Belgium, Germany, and Poland showed levels of total GSLs between 1270–3060 mg kg^−1^ DW [37]. On the other hand, Spanish Hinova, Megaton, Alfredo, Candella, and Bronco cabbage cultivars, Heckla and Predikant white cabbage cultivars, and Early Round Dutch *B. oleracea* cv. contained higher respective levels of GSLs of 2862–7949, 5087–5803, and 6995–18045 mg kg^−1^ DW [6,38,39], respectively.

The growth under ecological conditions increased the content of GSLs in Target and Albatros cv. (Table 1). The infection with *A. brassicicola* also increased the content of total GSLs in both varieties. In comparison, an increase of total GSLs resulting from fungal infections by *L. maculans* or *Fusarium oxysporum* was observed in *B. rapa* Herfstraap cv. but not in the Oleifera cv. [34]. A similar increase was induced by root colonization of *Arabidopsis thaliana* by *T. asperelloides* [32].

### 2.3. GSL Composition Profiles of Albatros and Target cv.

Table 2 shows a list of indol and aliphatic GSLs detected in samples of Albatros and Target cv. Glucobrassicin, 4-hydroxyglucobrassicin, and methoxyglucobrassicin/neoglucobrassicin belong to the group of indole GSLs whereas the others belong to the group of aliphatic GSLs. The GSL profile of both cultivars is typical for cabbage with a majority of indole and aliphatic GSLs present [16]. Concerning the latter group, molecules with propyl-, butyl-, 4-methylsulfonylbutyl-, 4-methylthiobutyl-, and 5-methylthiopentyl side-chains were missing. On the other hand, the compounds with 4-methylsulfinylbutyl- and 4-methylsulfinylbut-3-enyl described in broccoli and radish, respectively, were detected in Albatros and Target cv. [16]. No presence of aromatic GSLs, such as glucotropaeolin and gluconasturtin, was found in our cabbage samples.

The major GSLs present in Albatros and Target cv. in the amounts ≥200 mg kg^−1^ DW were glucobrassicin and sinigrin, the contents of glucoiberin were between 100 and 200 mg kg^−1^ DW (Table 3 and Table 4). The presence of glucobrassicin and sinigrin was discussed in connection with the resistance to fungal infections [34,40]. The amount of progoitrin in Albatros exceeded a value of 100 mg kg^−1^ DW only in the infected cabbage samples (Table 3 and Table 4), and its amount in Target cv. was lower (Table 4). Methoxylated indolic GSLs, 4-methoxyglucobrassicin, and neoglucobrassicin, were present in slightly higher amounts than other minor GSLs (Table 3 and Table 4). A comprehensive survey of *Brassicaceae* plants mentioned glucobrassicin as the predominant indole GSL making 60% of the indole GSL fraction in shoots. In roots, methoxy derivatives dominated, glucobrassicin represented only 23% [14]. Table 3 and Table 4 document this dominant position of glucobrassicin also in our cultivars where it represented 70–80% of the indole GSLs measured.

When Albatros and Target cv. obtained in the noninfected integrated vs. noninfected ecological production systems were compared with respect to their content of GSLs, the respective detected levels were 982 vs. 1014 and 798 vs. 916 mg kg^−1^ DW (Table 1). The statistical analysis of the results by ANOVA indicated increased GSL contents in the ecological production system. This finding probably reflected an activation of the plant defense system due to the lack of exogenous protection by pesticides. Both cultivars showed an increase in the content of methoxylated indole GSLs when produced in the ecological system, which is in agreement with the putative role of methoxylated indole GSLs in plant defense [32]. The changes in the concentration of other major GSLs, sinigrin, glucobrassicin, and glucoiberin were different in the two cultivars tested (Table 3 and Table 4). In Target cv., the growth under ecological conditions resulted in an increase of gluconapin (3.7-fold) and progoitrin (3.2-fold) (Table 4). The fungal infection by *A. brassicicola* resulted in a 30–50% increase of GSLs in both cultivars, with the exception of Albatros cv. grown in the integrated system (Table 3 and Table 4). This finding is in keeping with recent studies of fungal infections [16,41]. Major GSLs contributed to the infection-dependent increases, namely sinigrin, glucobrassicin, progoitrin, and glucoiberin, but the trends observed were often contradictory and dependent on the production system or the cultivar. Sinigrin was implicated in the resistance to the fungus *Peronospora parasitica* [40]. A 1.2–1.3-fold increase in the content of methoxylated indole GSLs, 4-methoxyglucobrassicin, and neoglucobrassicin was observed in both cultivars except for Albatros grown in the integrated system. This observation confirms the findings obtained with *B. rapa* exposed to *A. brassicicola* and *B. cinerea* [42] and is in accordance with the hypothesis on the role of methoxy indole GSLs in plant defense [14].

### 2.4. Degradation Products of the Spontaneous Enzymatic Hydrolysis of GSL

**Figure 1:**
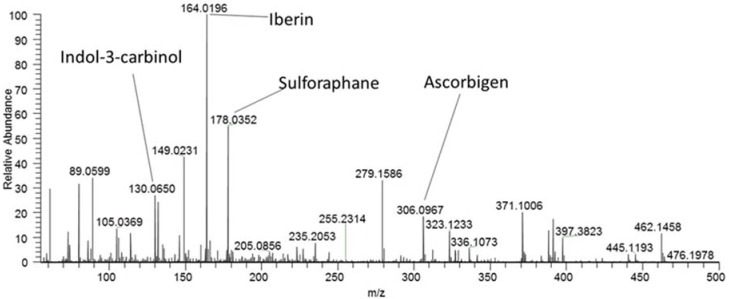
DART-Orbitrap-MS spectrum of an ethylacetate extract of Albatros cv. (integrated production) measured in the positive ionization mode. The identified GSL degradation products are described in Table 5.

Degradation products resulting from the enzymatic hydrolysis of GSLs by myrosinases have broad anticarcinogenic, antimicrobial and other beneficial effects on human health [43,44]. Four compounds were identified in our cabbage samples after homogenization and extraction with ethylacetate, namely sulforaphane, iberin, indole-3-carbinol, and ascorbigen (Table 5, Figure 1). They were produced by hydrolysis of glucoraphanin, glucoiberin, and glucobrassicin detected in the cabbage tissue (cf. Table 2) by inherent myrosinases after homogenization [45]. Iberin, indol-3-carbinol, and ascorbigen were described as major breakdown GSL products in white cabbage, and they are, also, found in other *Brassica* vegetables such as broccoli, cauliflower, and Brussels sprouts [44,46]. Ascorbigen is formed by a spontaneous reaction of indole-3-carbinol, originating from the enzymatic hydrolysis of glucobrassicin, with L-ascorbic acid. The amount of ascorbigen in homogenized white cabbage is equal to that in cauliflower and exceeds those detected in Chinese cabbage and broccoli [47]. The occurrence of sulforaphane was, also, reported in broccoli, Brussels sprouts, cauliflower, and some cabbage cv. [48]. Albatros and Target cv. are shown to be an important source of GSLs, and their degradation products enrich the human diet with the GSL-based, health-beneficial compounds. These molecules were implicated in protection against various types of cancer, high blood pressure reduction, heart disease prevention, and the control of blood glucose level to help in the type 2 diabetes [44,48].

### 2.5. Effect of Production Systems and Fungal Infection on Albatros and Target cv. Yields

**Figure 2:**
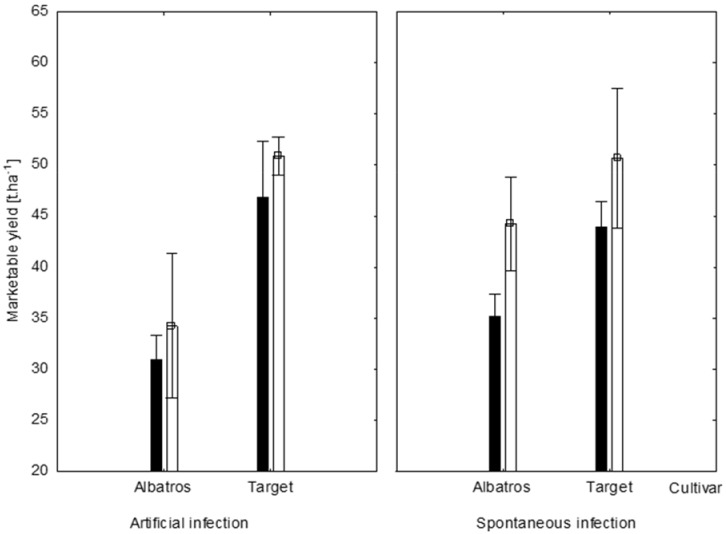
Effects of production system and fungal infection on the crop yield of Albatros and Target cultivars. Integrated system (unshaded columns), ecological system (shaded columns).

The yield of Target cv. was slightly higher than that of Albatros cv. in both production systems (Figure 2). The artificial infection with *A. brassicicola* did not significantly decrease the yield of cabbage heads probably because the disease severity measured as the proportion of foliage affected by the artificial infection, compared to the spontaneous infection, was relatively small, exceeding the spontaneous infection values by only 7 to 12% (Table 1). The reduction of plant green leaf area due to the artificial infection was evidently too small to significantly reduce photosynthesis [49]. No important effect of the type of production on the cabbage biomass yield was observed (Table 1, Figure 2).

## 3. Materials and Methods

### 3.1. Biological Material and Chemicals

Albatros F1 and Target F1 cabbage cv. (*Brassica oleracea* convar. *capitata* (L.) Alef. var. *capitata* f. *alba*) were obtained from Moravoseed a.s. (Mušlov, Czech Republic) where they were bred. Albatros is a hybrid, mate-ripening variety of cabbage intended for storage, having a medium-size, solid, and tight head of average fresh weight of 2.2–2.8 kg. Target is a hybrid, semilate variety of cabbage bred for the production of sauerkraut and use as the fresh vegetable. It is characterized by round, solid, tight heads of an average fresh weight of about 3.3 kg. Both varieties are exported and widely used in eastern Europe.

Fungal pathogen *Alternaria brassicicola* (Schwein.) CCF 2749 was obtained from the Culture Collection of Fungi (Charles University, Prague, Czech Republic) and maintained on potato dextrose agar slants (Difco, USA) and reinoculated every 2–3 months.

Methanol, acetonitrile, and ethyl acetate were LC-MS purity (Honeywell, Offenbach, Germany). Deionized water (18 MΩ cm) was produced by a Milli-Q system (Millipore, Bedford, MA, USA). Ammonium formate (>99%) for preparation of LC mobile phase was purchased from Sigma-Aldrich (Steinheim, Germany). Meta-Phosphoric and ortho-Phosphoric acids in analytical grade were obtained from Penta (Chrudim, Czech Republic).

### 3.2. AA Analysis

AA content in biomass was measured using a modified method of Lundegardh et al. (2008) [50]. To obtain a representative sample preparation longitudinal slices of an approximative weight of five grams made from four cabbage samples were mixed, and the total biomass of 20 g was extracted with metaphosphoric acid (30 g L^−1^) during homogenization in a laboratory blender at the room temperature. AA was analyzed by an HPLC method with DAD detection (liquid chromatograph HP 1200 with DAD detector, Agilent Technologies, Santa Clara, CA, USA). The conditions were: LiChroCART, LiChrospher 100 RP-18 (Merck, Darmstadt, Germany) chromatographic column (125 × 4 mm id., 5 μm) with precolumn (4 × 4 mm id., 5 μm); mobile phase 5% methanol (*v*/*v*), pH = 3 (H_3_PO_4_); flow 0.8 mL min^−1^; temperature 35 °C; injection volume 5 μL; UV detection at 244 nm.

The identification of AA in the samples was carried out by comparing the retention time with that of the standard (L-ascorbic acid, Sigma Aldrich, Steinheim, Germany, purity ≥99%). For quantification, an external calibration curve was used. The method characteristics were the following: repeatability expressed as RSD 5%, recovery 95%, and LOQ 0.15 mg kg^−1^ DW.

The cabbage dry mass was obtained by drying at 105 °C for 5 h.

### 3.3. GLS Analysis

A sample (20 g) taken from an intact part of the plant was immediately added to 70% methanol (*v*/*v*) and homogenized in a laboratory blender. The extract was filtered through a membrane filter (0.45 μm) into a vial and analyzed. The analyses were performed using the UHPLC system Acquity UPLC^®^ (Waters, Milford, MA, USA) coupled with Orbitrap mass spectrometer ExactiveTM (Thermo Fisher Scientific, Germany). The LC separation was performed by an Atlantis HILIC Silica column (150 mm × 2.1 mm i.d., 3.0 μm), (Waters, Milford, MA, USA). The mobile phase consisting of (A) acetonitrile and (B) 10 mM ammonium formate was used for gradient elution. The Orbitrap mass spectrometer was operated in both the negative and positive electrospray ionization (ESI) mode. The parameter settings used during the measurements were as follows: capillary voltage (±700 V), cone voltage (±25 V), source temperature (120 °C), and desolvation temperature (350 °C). Nitrogen was used as both desolvation and cone gas at a flow rate of 800 and 10 L h^−1^, respectively. Full scan MS spectra were acquired in a range of *m*/*z* 50–1000.

Individual GSLs were identified according to their exact masses, for semi-quantification of the detected compounds, sinigrin [(−)-sinigrin hydrate, Sigma Aldrich, Steinheim, Germany, purity ≥99%] was used as the external standard. The results represent an average of four parallel measurements.

The method characteristics were the following: repeatability expressed as RSD 4%, LOQ 0.003 mg kg^−1^ DW.

### 3.4. Degradation Products Analysis

DART-Orbitrap-HRMS system consisted of DART-SVP ion source (IonSense, Saugus, MA, USA) with an XZ transmission module autosampler (IonSense, Saugus, MA, USA) coupled to the Orbitrap mass spectrometer ExactiveTM (Thermo Fisher Scientific, Bremen, Germany). Vapur interface (IonSense, Saugus, MA, USA) was employed to hyphenate the ion source and the mass spectrometer. Methanolic and ethylacetate cabbage extracts were measured in both the positive and negative ionization mode. The former extract measured in the negative ionization mode was most suitable for detection of polar compounds whereas the latter measured in the positive ionization mode for detection of the nonpolar ones. The preparation of samples was the following: The samples were homogenized, kept at room temperature, and 1 h after the homogenization extracted with the solvent by 2-min intense shaking (cabbage sample homogenate 5 g; solvent 5 mL). Then the solid phase was removed by centrifugation (10,000 rpm, 5 min). The operation parameters of the ion source and mass spectrometer were optimized. The final parameters and conditions of the DART-Orbitrap-HRMS analysis are seen in Table 6.

The methanolic extracts measured in the negative ionization mode detected mostly organic acids, such as succinate and malonate, and saccharides. The ethylacetate extracts measured in the positive ionization mode showed decomposition products of GSLs predominantly.

### 3.5. Agricultural Production Systems

The integrated system was characterized by mineral fertilization [180 kg nitrogen (N) ha−1] and insecticide protection. The fertilization was applied in two steps, 108 kg N ha^−1^ of the total amount was added before the cabbage plants were planted and 72 kg N ha^−1^ at the phase of the eighth primary leaf. The insecticide treatment included the addition of pyridate (Lentagran, Belchim Crop Protection, Londerzeel, Belgium, 1 kg ha^−1^) and deltamethrin (Decis Mega, Bayer, Germany, 0.1 L ha^−1^) on 19th June 2013, lambda-cyhalothrin (Karate with Zeon technology 5CS, Syngenta, Basel, Switzerland, 0.2 L ha^−1^) on 1st July 2013, and deltamethrin (Decis Mega, Bayer, Germany, 0.1 L ha^−1^) on 22nd August 2013.

The ecological system using ecological plots certified in keeping with the Czech legislation was characterized by using only organic fertilization (Organica N pelleted fertilizer produced from poultry bedding, molasses and molasses ale, Agro CS, Česká Skalice, Czech Republic) and by the absence of chemical protection. The fertilizer was applied at an amount of 3 tons ha^−1^ before the cabbage plants were planted. No insecticide was used in this production system.

The experiments were conducted at Czech University of Life Sciences experimental station in Troja (altitude 195 m, modal fluvisol soil, pH 6.9). The experimental design included four parallel randomized blocks, each representing 12 plants, using three factors, namely, production system, fungal infection and the cultivar. The climatic conditions monitored in the year when the experiments were conducted were compared to the 30-year average temperature and precipitation profiles of the site: Except for June, the temperature was above the 30-year average values, the amount of precipitation was decreasing in the period of May-June and then, remained below the 30-year average level (cf. Supplementary Materials).

### 3.6. Fungal Infection and Disease Assessment

The spore inoculum of *A. brassicicola* was prepared according to a modified method of Strandberg (1977) [51]. The fungus was grown on the vegetable juice V8 (Campbell Soup Co., Camden, NJ, USA) agar medium (vegetable juice 10-fold diluted with distilled water, agar 20 g L^−1^, pH 6) in Petri dishes inoculated with 0.5 × 0.5 cm agar blocs covered with a fungal mycelium from 2-week-old V8 agar cultures. The agar cultures were incubated for two weeks at 24 °C in the dark at a high humidity to ensure sporulation. A spore suspension was prepared using sterile distilled water containing Triton X-100 (100 μL L^−1^), filtered to remove the cell debris, and adjusted to a concentration of 5 × 10^5^–1 × 10^6^ spores in 1 mL that was used for inoculation of plants by atomizer at a volume of 5 mL per plant [51,52].

Albatros and Target cv. were sown and the plants pregrown in a greenhouse at 18–20 °C for one month and planted to an experimental field (plant spacing, 50 × 60 cm). The spore suspension was applied to 2-month-old plants, and they were kept under a propylene textile cover for three days to maintain a high humidity. Then the cover was removed and the plants grown in outdoor conditions for five months. The disease severity was expressed using an arbitrary scale (cf. Table 1). Triplicates were used in all experiments, and the infected plants were compared with the noninfected controls.

### 3.7. Statistical Evaluation

Statistical analysis was carried out using the STATISTICA 12.0 software system (Stat Soft, Palo Alto, CA, USA). The data measuring the infection by fungal pathogen were analyzed by ANOVA statistical program with the subsequent application of Fisher’s LSD test (*p* ≤ 0.05).

## 4. Conclusions

Czech white-cabbage Albatros and Target cv. demonstrated AA and GSL contents comparable to those of various commercial varieties of white cabbage (*B. oleracea* var. *capitata*) used worldwide for cabbage production. No important effect of the ecological and integrated production systems on the dry weight biomass yields was found which ensured a stable crop yield under various production conditions. The total AA and GSL contents and profiles of the individual GSLs in both cultivars, including the presence of enzymatic-hydrolysis products sulforaphane, iberin, indole-3-carbinol, and ascorbigen, demonstrated a high-quality of the acknowledged human-health beneficial effects. Used in commercial production, Albatros and Target cv. have a potential to be an important source of valuable nutrients, antioxidants, and phytocompounds with anticancer properties in human nutrition.

## Figures and Tables

**Table 1:** Effect of production system and artificial fungal infection on the content of ascorbic acid (AA) and glucosinolates (GSLs) in Albatros and Target cultivars (cv.).

Cultivar	Production System	Disease Severity ^1^	Dry Biomass (%)	AA ± SD ^2^ (mg kg^−1^)	Total GSLs ± SD ^2^ (mg kg^−1^)
Albatros	Integrated	3.04a ^*^	10.8	4326 ± 216	911 ± 36
		2.71a ^**^	9.7	1713 ± 86	982 ± 39
	Ecological	3.63c ^*^	10.2	4275 ± 214	1470 ± 59
		3.38b ^**^	10.5	3795 ± 190	1014 ± 41
Target	Integrated	3.29b ^*^	8.7	2933 ± 147	1090 ± 44
		2.96a ^**^	8.6	3271 ± 186	798 ± 32
	Ecological	4.17e ^*^	9.2	2684 ± 134	1320 ± 53
		3.83c ^**^	9.1	3353 ± 168	916 ± 37

^1^ Disease severity is expressed using an arbitrary scale: 0 points—no visible disease damage, 1 point—few scattered lesions (<5% leaf area damaged), 3 points—5–30% leaf area damaged, 5 points—30–60% leaf area damaged, 7 points—60–90% leaf area damaged, 9 points—>90% leaf area damaged. One-asterisk superscript indicates the situation when the artificial infection with *A. brassicicola* was applied, two-asterisk superscript indicates the control when no infection was applied, and the values represent only the spontaneous infection. The statistical analysis used ANOVA and Fisher’s Least Significant Difference (LSD) test. Different letters indicate that the values are statistically different (*p* ≤ 0.05). ^2^ Contents of AA and GSLs are related to dry biomass. The figures represent the mean ± SD values.

**Table 2:** GSLs found in Albatros and Target cv.

Glucosinolate	Abbreviation	Formula	Ion Type	*m*/*z*
Glucobrassicin	GB	C_16_H_20_N_2_O_9_S_2_	[M − H]^−^	447.0532
4-hydroxyglucobrassicin	HGB	C_16_H_20_N_2_O_10_S_2_	[M − H]^−^	463.0481
Methoxyglucobrassicin/neoglucobrassicin	MGB/NGB	C_17_H_22_N_2_O_10_S_2_	[M − H]^−^	477.0638
Sinigrin	SINI	C_10_H_16_KNO_9_S_2_	[M − H]^−^	358.0267
Glucoiberin	IBER	C_11_H_21_NO_10_S_3_	[M − H]^−^	422.0249
Progoitrin	PROG	C_11_H_19_NO_10_S_2_	[M − H]^−^	388.0372
Glucoraphanin	RAPHA	C_12_H_23_NO_10_S_3_	[M − H]^−^	436.0406
Gluconapin	NAPI	C_11_H_19_NO_9_S_2_	[M − H]^−^	372.0423
Glucoibervirin	IBEV	C_11_H_21_NO_9_S_3_	[M − H]^−^	406.0300

**Table 3:** Effect of production system and fungal infection on the composition and amount of GSLs in Albatros cv.

System	SINI	GB	MGB/NGB	HGB	IBEV	NAPI	PROG	IBER	RAPHA	Total
I*	241	289	53	19	10	59	109	106	26	912
I**	188	464	78	21	7	32	63	108	22	982
E*	342	418	116	23	16	115	222	175	44	1470
E**	337	278	92	14	11	36	59	156	30	1014

I*, integrated infected system; I**, integrated noninfected system; E*, ecological infected system; E**, ecological noninfected system. GSL amounts were quantified as sinigrin equivalent and expressed in mg kg^−1^ cabbage dry biomass. Abbreviations: Sinigrin (SINI), glucobrassicin (GB), Methoxyglucobrassicin/Neoglucobrassicin (MGB/NGB), 4-hydroxyglucobrassicin (HGB), glucoibervirin (IBEV), gluconapin (NAPI), progoitrin (PROG), glucoiberin (IBER), glucoraphanin (RAPHA).

**Table 4:** Effect of production system and fungal infection on the composition and amount of GSLs in Target cv.

System	SINI	GB	MGB/NGB	HGB	IBEV	NAPI	PROG	IBER	RAPHA	Total
I*	294	507	98	13	8	20	34	107	9	1090
I**	267	260	73	15	8	15	26	119	14	798
E*	436	390	99	20	13	42	88	195	37	1320
E**	208	348	84	18	5	55	82	91	25	916

I*, integrated infected system; I**, integrated noninfected system; E*, ecological infected system; E**, ecological noninfected system. GSL amounts were quantified as sinigrin equivalent and expressed in mg kg^−1^ cabbage dry biomass. Abbreviations: Sinigrin (SINI), glucobrassicin (GB), Methoxyglucobrassicin/Neoglucobrassicin (MGB/NGB), 4-hydroxyglucobrassicin (HGB), glucoibervirin (IBEV), gluconapin (NAPI), progoitrin (PROG), glucoiberin (IBER), glucoraphanin (RAPHA).

**Table 5:** Degradation products of GSLs identified in Albatros and Target cv.

Degradation Products	Summary Formula	Ion Type	*m*/*z*
Sulforaphane	C_6_H_11_NOS_2_	[M + H]^+^	178.0360
Iberin	C_5_H_9_NOS_2_	[M + H]^+^	164.0204
Indole-3-carbinol	C_9_H_9_NO	[M + H − H_2_O]^+^	130.0657
Ascorbigen	C_15_H_15_NO_6_	[M + H]^+^	306.0978

**Table 6:** Optimized conditions used for DART-Orbitrap-MS analyses.

Ionization Mode	Positive	Negative
Extraction solvent	EtAc ^1^	MeOH ^2^
Ionization gas temperature (He)	450 °C	350 °C
Ionization gas pressure (He)	5.5 bar	5.5 bar
Desorption time	7 s	7 s
Capillary voltage	40 V	−50 V
Voltage of ion optical system	250 V	−150 V
Skimmer voltage	20 V	−20 V
Capillary temperature	250 °C	250 °C
Resolution	50,000 FWHM	50,000 FWHM

^1^ Ethylacetate (EtAc); ^2^ methanol (MeOH).

## Supplementary Materials

The 30-year, average temperature and precipitation profiles of the site are available as supplementary files online: Figures S1 and S2.
