# Supplementary material for: Ascorbic Acid and Glucosinolate Levels in New Czech Cabbage Cultivars: Effect of Production System and Fungal Infection
Source: Molecules. 2018 Jul 25;23(8):1855. doi: 10.3390/molecules23081855 (PMC6222616; doi:10.3390/molecules23081855)
Supplement: Supplementary file 1 [file molecules-23-01855-s001.pdf]

Supplementary Material

Climatic conditions at the experimental agrostation during cabbage-growing period

The experiments were conducted at the experimental station of Czech University of Life Sciences, Prague in Troja (altitude of 195 m above sea level). The locality can be characterized as a dry, medium-warm one with a soil classified as modal fluvisol (pH 6.9). The climatic conditions monitored in the year 2013 and compared to the 30-year, average temperature and precipitation profiles of the site are shown in Figs. 1 and 2. Except for June the temperature was above the long-term average values. The amount of precipitation was decreasing in the period of May to June 2013 and then remained below the long-term average level.

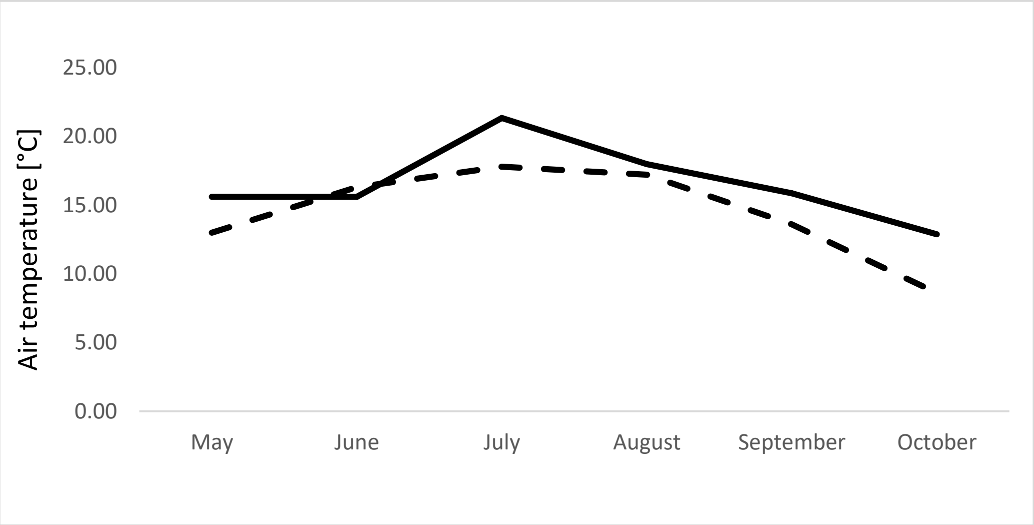

Figure S1. Air temperature profile at the Troja trial station during the growing season of 2013 (solid line) compared to the 30-year average temperature profile of the site (dashed line).

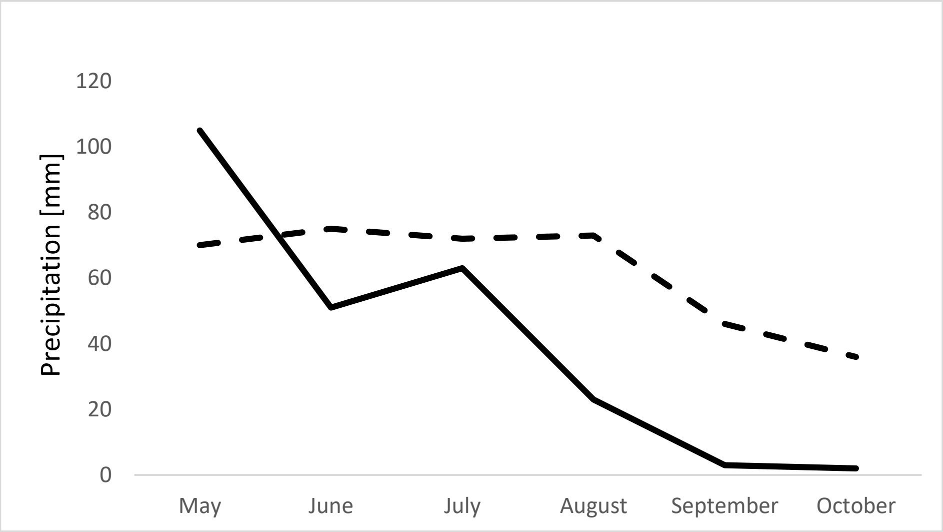

Figure S2. Precipitation profile at the Troja trial station during the growing season of 2013 (solid line) compared to the 30-year average precipitation profile of the site (dashed line).
